# Case Report: A giant but silent adrenal pheochromocytoma – a rare entity

**DOI:** 10.12688/f1000research.8168.1

**Published:** 2016-03-07

**Authors:** Sunil Munakomi, Saroj Rajbanshi, Prof Shailesh Adhikary

**Affiliations:** 1 Department of General Surgery, B.P. Koirala Institute of Health Sciences, Dharan, Nepal

**Keywords:** Giant, benign, silent, pheochromocytoma

## Abstract

Herein we report a rare entity of a giant adrenal pheochromocytoma in a fifty-year-old male presenting with a vague abdominal pain. A computerised tomogram of the abdomen revealed a well-defined  left supraadrenal giant lesion with no evidence of invasion to surrounding structures.The patient underwent surgical excision without any untoward postoperative events. Histopathological study revealed a benign pheochromocytoma. This report highlights the importance of acknowledging the fact that sometimes a giant adrenal pheochromocytoma can present with paucity of clinical  signs and symptoms.Thorough investigations and a multidisciplinary team approach may lead  to a better outcome in these patients.

## Introduction

Giant pheochromocytomas (> 7 cm in size) are rare entities with around 20 cases reported in the literature ^1– 6^. They do not present with the classical symptoms of pheochromocytomas ^6^. Most patients present with vague discomfort while others may complain of a palpable abdominal mass. Operative surgery is the ideal management option ^7^. There needs to be a multidisciplinary approach while managing such cases. Stringent preparation to combat crisis due to catecholamine surge (during tumor manipulation) and sudden decrease in peripheral vascular resistance (following lesional excision) need to be emphasized ^8, 9^. Presence of chromaffin cells in the extra-adrenal tissue is the only confirmative method of distinguishing the malignant variant from its benign counterpart ^10^. Herein we highlight and discuss the management algorithm taken while managing one such case.

## Case report

**Figure 1.**
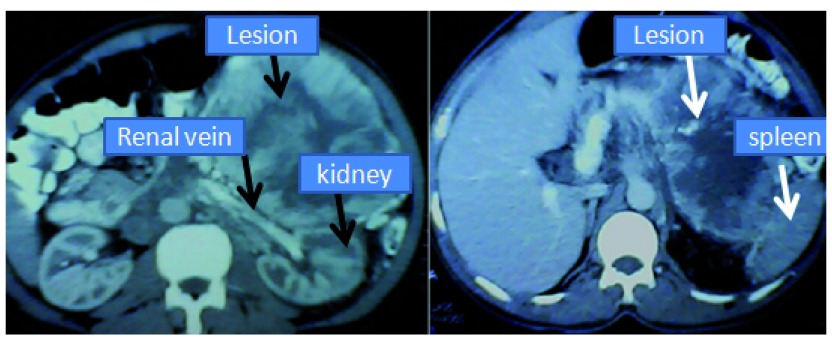
CT images showing the giant suprarenal lesion abutting the spleen but with no invasion of the kidney or the renal vein.

A fifty-year-old male from Dhahran, Nepal presented to the surgical outpatient clinic with a vague symptom of abdominal discomfort. He had no history of trauma, persistent vomiting, altered bowel habits, change in the color of the stool or abdominal distension. There were no significant past medical or surgical illnesses. Family history of similar symptoms was also absent. Examination of the abdomen was normal except for slight discomfort during palpation in his left upper quadrant. Ultrasound examination of the abdomen revealed a huge left suprarenal mass. Computerised tomogram (CT) of the abdomen confirmed a giant mass of approximately 12×8 cm ^2^ in the left suprarenal region showing rim enhancement and areas of low attenuation within it. The left renal vein was normal and the lesion was slightly abutting the spleen ( Figure 1).

The patient denied attacks of headache, cheat pain, palpitation and sweating. The serum and urinary catecholamine levels were within normal range. The patient was kept for observation with 24 hour electrocardiography (ECG) and blood pressure monitoring (BP) which was normal.

**Figure 2.**
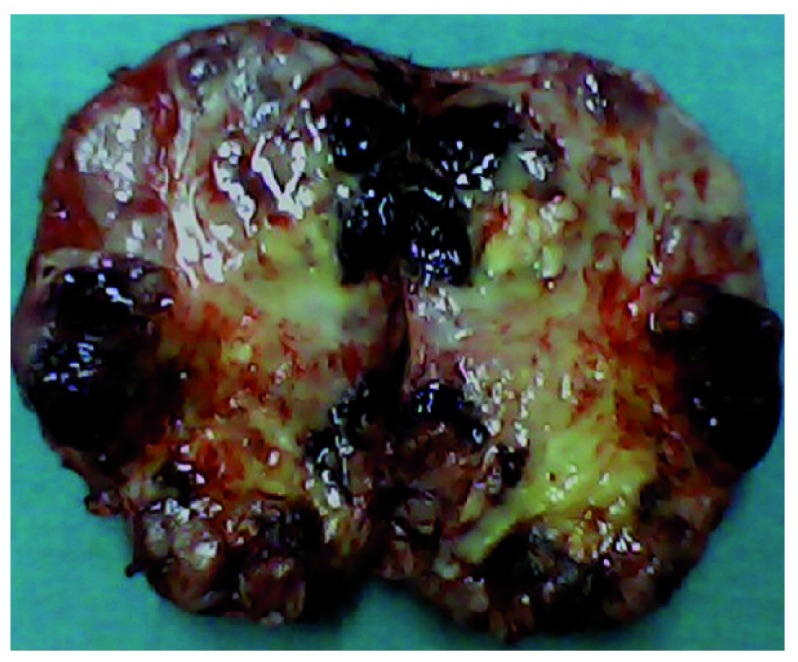
Cut specimen of the excised lesion showing areas of scattered hemorrhages.

The patient and his relatives were explained of the disease entity and were advised for surgery. With written consent, he was prepared for surgery. The anesthesiologists prepared medication (Intravenous (i.v.) Phentolamine (1 mg injection), Nitroprusside (4 mg drip) and Esmolol (30 mg injection) for potential intra-operative crisis pertaining to catecholamine surge during surgical manipulation. Vasoactive agents were also made available for combating sudden loss of peripheral vascular resistance following tumor removal. Early vascular control was secured. There was a well demarcated plane to dissect the tumor from the surrounding structures ( Figure 2).

**Figure 3.**
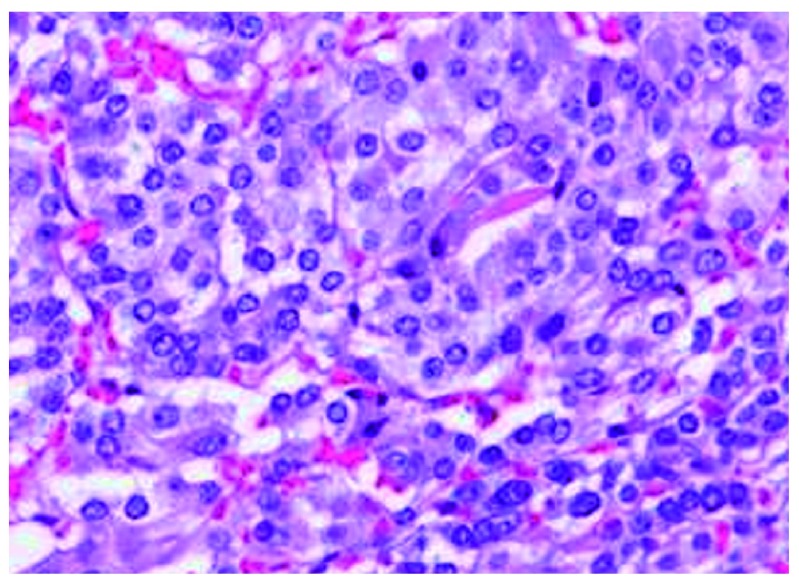
Histopathology revealing characteristic zellballen nests of cells separated by fibro vascular stroma.

The patient was extubated and was kept in the intensive care unit (ICU) for 48 hours. There were no untoward events in the post-operative period and the patient was discharged home on the 10 ^th^ day. Histopathological study revealed zellballen nests of chromaffin cells with no invasion of the capsule ( Figure 3), which is highly suggestive of a benign pheochromocytoma.

The patient is asymptomatic 4 years following surgery, and has been advised to follow up periodically in order to rule out early recurrence.

## Discussion

Pheochromocytomas typically present with the characteristic triad of paroxysmal attacks of headache, sweating and palpitation ^11^. However giant lesions paradoxically may lack these symptoms ^6^. The reasons for the same can be due of the presence of tumoral necrosis, high loads of interstitial tissue compared to chromaffin cells or the paucity of the release of the catecholamines due to encapsulation by the connective tissues ^6^. This may also cause normal catecholamine values during their serum and urinary assays ^2^.

CT scan is the image modality of choice to diagnose the condition ^4^. However, in cases of giant lesions, there may be difficulties indetermining the organ of origin o leading to mis-diagnosis of the entity ^2^.

Open surgical removal is the therapeutic target ^7^. Laparoscopic removal is reserved only for smaller lesions ^12, 13^. Some authors have suggested preoperative embolisation of theses lesion ^14^. However, it may be tenacious due to major arterio-venous connections within the lesion ^6^. The key to a successful outcome is the fine tuning between the surgeons and the anesthesist in the peri-operative period ^8, 9^. There needs to be minimal handling of the lesion and an early control of the adrenal vein so as to limit crisis due catecholamine surge ^6^.

Pheochromocytoma of the adrenal gland scaled score (PASS) score has been described to differentiate between the benign and the malignant lesions ^15^. But the hallmark of the malignant counterpart is the presence of the ectopic chromaffin cells in the extra-adrenal sites ^10^.

The patients need to be on a periodic follow up so as to exclude the risk of recurrence ^16^. There are still no set therapeutic guidelines in the management of the malignant lesions due to paucity of cases. Long term prognosis is dismal with five year survival of around 50% only ^17^.

## Conclusion

Though benign, surgery is advocated for giant pheochromocytomas. Early vascular control, minimal handling of the tumor and a multidisciplinary approach to combat potential intra-operative crisis are the cornerstones in managing such cases. Malignant counterparts need to be excluded histologically. Patients require regular follow up to rule out recurrence.

## Consent

Written informed consent was obtained from the patient for publication of this case report and any accompanying images and/or other details that could potentially reveal the patient’s identity.
