# Supplementary material for: Case Report: A giant but silent adrenal pheochromocytoma – a rare entity
Source: F1000Res. 2016 Mar 7;5:290. [Version 1] doi: 10.12688/f1000research.8168.1 (PMC5022706; doi:10.12688/f1000research.8168.1)
Supplement: Peer review history

# Peer review: Referee response for version 1

**DOI:** 10.5256/f1000research.8785.r16138

**Published:** 2016-09-12

**Authors:** Prasanna Kumar Reddy, Nadeem Mushtaque, Vimalkumar Dhaduk

**Affiliations:** 1 Department of Minimal Access Surgery, Apollo Hospitals, Chennai, India

The format of the paper and the language are good. Giant adrenal tumors are not that rare, but always pose a diagnostic dilemma.

 The CT pictures are informative and remaining diagnostic work up was done well. Even though it is solitary, non functioning, and showing no evidence of capsular invasion or any distant metastasis, a lot of emphasis was given on malignancy. The size of the tumor does not have any significance on malignancy. However one has to be aware of malignant potential.

 The technique was described well, but the importance of not breaching the capsule was not sufficiently emphasized. Intraoperative hemodynamic parameters were not highlighted.

 Presently the procedure of choice is laparoscopic adrenalectomy even up-to the size of 12 cm in minimally invasive surgical units with expertise as left supra renal is easily amenable. Vascular control with the presently available gadgets is much easier and the manipulation of the tumors is very minimal.

 Corrections:

- Abstract - supra adrenal gland was mentioned instead of supra renal gland.
- Case report - Line 9 instead of chest pain it is misspelled as cheat pain.

The conclusion, even though it is adequate, is too brief.

 An adequate number of references were quoted.

 Impression - A good paper contributing various facts of giant pheochromocytoma, even though it is a single case report.

We have read this submission. We believe that we have an appropriate level of expertise to confirm that it is of an acceptable scientific standard.

# Peer review: Referee response for version 1

**DOI:** 10.5256/f1000research.8785.r13171

**Published:** 2016-04-22

**Authors:** Venugopal Sarveswaran

**Affiliations:** 1 Department of General Surgery, Sri Ramakrishna Hospital, Coimbatore, India

I feel the case report seems to be an original one, though such cases have been published previously.

It provides sufficient details for others to learn, like it emphasizes that such clinical condition must be kept in mind and adequate safety precautions must be taken while operating on such patients, though all cases will not have intra-operative complications.

It states the clinical condition and investigations done.

An adequate number of references have been sited.

Hence I feel the article is acceptable.

I have read this submission. I believe that I have an appropriate level of expertise to confirm that it is of an acceptable scientific standard.
